# An overview of comparative modelling and resources dedicated to large-scale modelling of genome sequences

**DOI:** 10.1107/S2059798317008920

**Published:** 2017-07-28

**Authors:** Su Datt Lam, Sayoni Das, Ian Sillitoe, Christine Orengo

**Affiliations:** a Institute of Structural and Molecular Biology, UCL, Darwin Building, Gower Street, London WC1E 6BT, England; b School of Biosciences and Biotechnology, Faculty of Science and Technology, University Kebangsaan Malaysia, 43600 Bangi, Selangor, Malaysia

**Keywords:** protein structure prediction, template-based modelling, comparative modelling, template selection

## Abstract

Computational modelling of proteins has been a major catalyst in structural biology. Bioinformatics groups have exploited the repositories of known structures to predict high-quality structural models with high efficiency at low cost. This article provides an overview of comparative modelling, reviews recent developments and describes resources dedicated to large-scale comparative modelling of genome sequences. The value of subclustering protein domain superfamilies to guide the template-selection process is investigated. Some recent cases in which structural modelling has aided experimental work to determine very large macromolecular complexes are also cited.

## Abstract 2

This paper reviews the recent advances in computational template-based structural modelling and proposes the subclustering of protein domain superfamilies to guide the template-selection process.

## 1. Introduction   

In May 2017, the Protein Data Bank (PDB; Berman *et al.*, 2000 ▸) celebrated a milestone release of 130 000 entries. There is still a steady flow of new structures, with more than 100 added each week. However, there remains an ever-widening gap between sequence and structure space, with more than 85 million protein sequences currently deposited in the UniProtKB/TrEMBL database (The UniProt Consortium, 2017 ▸). Thanks to structural genomics initiatives (Nair *et al.*, 2009 ▸; Terwilliger, 2011 ▸; Schwede, 2013 ▸), which have deliberately solved the structures of structurally uncharacterized families, there are increasing numbers of sequences for which there are homologues of known structure. Various protein structure modelling approaches have been developed. In this review, we focus on comparative modelling.

## 2. Comparative modelling   

The most commonly used and most accurate protein structure modelling method is comparative modelling, which predicts the structure of an unknown protein using known information from one or more homologous partners. Comparative modelling usually involves three steps: (i) the identification of template structures for modelling the query protein, (ii) sequence alignment between the template and the query, and (iii) modelling the structure of the query.

### 2.1. Template-selection methods   

#### 2.1.1. Sequence-based methods   

Generally, all of the template-selection methods involve searching for template protein structures from the PDB. Global sequence identity between the query and templates has been used extensively as the primary criterion in a search process using *BLAST* (Altschul *et al.*, 1990 ▸). *BLAST* aligns two sequences based on a substitution matrix, the scoring scheme used to align two amino acids. A substitution matrix captures the probability with which a specific amino-acid residue mutates to/substitutes for another over a long period of evolutionary time.

Comparative modelling generally produces a good three-dimensional model if a homologous template with a global sequence identity of ≥30% is used. However, once the sequence identity falls below 30% (the ‘twilight zone’), the model quality deteriorates rapidly (Baker & Sali, 2001 ▸). *BLAST* treats the positions that tend to be conserved or variable in a protein family with the same weight, so that the signal becomes weak with distant homologues.

#### 2.1.2. Profile-based methods   

Sequence profiles that manage to capture the pattern embedded in a multiple sequence alignment of evolutionarily related relatives improve the sequence signal for template searching and alignment of the query with the template. Evolutionary information from homologous proteins was originally captured in position-specific scoring matrices (PSSMs). For example, *PSI-BLAST* (Altschul *et al.*, 1997 ▸) uses a PSSM to score matches between query and database sequences and is about three times more sensitive than *BLAST*.

Hidden Markov models (HMMs) are more advanced forms of sequence profiles. The revolutionary feature of HMMs is their ability to additionally capture the insertions and deletions that are found in a multiple sequence alignment. In addition, HMMs can also include predicted secondary-structure information in the profile. *HHsearch* (Söding, 2005 ▸) and *HMMER* (Eddy, 2011 ▸) are among two of the most popular HMM-based methods. These approaches have the ability to extend the sequence search into the ‘twilight zone’ and find templates which have high structural similarity to the query despite low global sequence identity. *Robetta* (Kim *et al.*, 2004 ▸; Ovchinnikov *et al.*, 2017 ▸), *BioSerf* (Buchan *et al.*, 2013 ▸), *SWISS-MODEL* (Biasini *et al.*, 2014 ▸), *nns* (Joo *et al.*, 2016 ▸) and *MULTICOM* (Li *et al.*, 2015 ▸) are examples of robust modelling servers that use HMM approaches to search for structural templates.

A more advanced form of sequence profile named conditional random fields (CRF) has also been proposed (Lafferty *et al.*, 2001 ▸). The main advantages of using CRFs over HMMs is the relaxation of the residue-independence assumptions that are required by HMMs (for further explanation, see Tang *et al.*, 2013 ▸). CRFs have been applied to various bioinformatic studies (Zhao *et al.*, 2010 ▸; Tang *et al.*, 2013 ▸; Ma & Wang, 2015 ▸; Joo *et al.*, 2016 ▸).

#### 2.1.3. Other considerations during template selection   

Various studies have highlighted the importance of considering the physical and structural environment of the template selected for modelling a particular query sequence such as pH, temperature, space group and quaternary structure (Fiser, 2004 ▸). However, Sadowski and Jones concluded that these factors do not significantly improve template selection for single-domain modelling (Sadowski & Jones, 2007 ▸). If there is more than one potential template with comparable sequence identity, it is preferable to use the template with the best X-ray resolution, regardless of conditions.

It is also possible to use multiple structural templates in the modelling process, especially for multi-domain protein modelling (Cheng, 2008 ▸; Meier & Söding, 2015 ▸). The inclusion of additional templates can improve the model quality, particularly by extending the coverage of the query sequence (Larsson *et al.*, 2008 ▸) or when the templates are structurally complementary (Chakravarty *et al.*, 2008 ▸). Multiple templates also provide conserved distance constraints, which are not available to single-template protocols (Cheng, 2008 ▸). However, if the templates are too diverse (*i.e.* contradictory) this can affect the quality of the model produced (Chakravarty *et al.*, 2008 ▸; Tress, 2013 ▸).

### 2.2. Sequence–template alignment   

Once a structural template has been identified, both the template and alignment (usually obtained from the template-searching method) can be submitted to a comparative modelling program to predict the three-dimensional atomic coordinates of the query protein. Overall, it is generally agreed that profile-based alignments produce better quality models than sequence-based alignments (Yan *et al.*, 2013 ▸). In addition, HMM-based alignments produced by *HHsearch* tend to give higher quality models than PSSM-based alignments produced by *PSI-BLAST* (Yan *et al.*, 2013 ▸).

Structural information has also been explored to produce a better sequence alignment, especially for multiple template-modelling and threading protocols (Pei *et al.*, 2008 ▸; Di Tommaso *et al.*, 2011 ▸; Daniels *et al.*, 2012 ▸). Threading protocols work by aligning the target sequence against protein-fold templates from known structures and evaluating how well the query aligns with the fold. A typical protein-fold library is compiled from protein structure databases such as CATH (Dawson *et al.*, 2017 ▸), SCOP (Andreeva *et al.*, 2014 ▸) and ECOD (Cheng *et al.*, 2015 ▸). The scoring functions commonly used capture secondary-structure match, residue–residue contacts and profile–profile alignment scores. In addition, composite scoring functions including multiple structural features (for example solvent accessibility and torsion angles) are also deemed to be useful (Wu & Zhang, 2008 ▸; Yang *et al.*, 2011 ▸). Subsequently, the best-fit alignment is usually generated with the help of dynamic programming. Some commonly used methods are *LOMETS* (Wu & Zhang, 2007 ▸; Yang *et al.*, 2015 ▸), the *THREADER* suite of methods (Lobley *et al.*, 2009 ▸; Buchan & Jones, 2017 ▸), *SPARKS-X* (Yang *et al.*, 2011 ▸) and *Raptor-X* (Ma *et al.*, 2013 ▸).

### 2.3. Modelling the structure   

In 1993, Andrej Sali and Tom Blundell developed *MODELLER*, which remains one of the most widely used comparative modelling methods (Sali & Blundell, 1993 ▸). The major steps in modelling the structure of a query sequence, based on a template structure, are summarized below. For a more detailed account, see the recent reviews by Saxena *et al.* (2013 ▸) and Tress (2013 ▸). Guided by the sequence–template alignment, comparative modelling methods usually start by copying the coordinates (structurally conserved regions) from the template to assemble the basic backbone of the model.

Processing deleted residues between the query and template sequence involves the removal of residues and closure of the hole formed by creating the new peptide bond. For insertions, loop modelling can be performed by searching through high-resolution fragment libraries (either derived from the PDB or structural domain resources such as CATH or SCOP) to find segments that fit the specific part of the backbone. However, these methods are limited by the fact that the number of possible conformations increases exponentially with the length of a loop (and become difficult when the loop size is >7 residues). By contrast, conformational approaches construct loops by searching through the conformational space of possible loop conformations driven by satisfying a specific energy function (for example stereochemical, distance or steric constraints). In order to maximize the accuracy of loop prediction, simulating the correct environment (energy functions) is key. Approaches to perform this include hybrid methods which employ both knowledge-based and physics-based energy functions (for more details, see, for example, Park *et al.*, 2014 ▸), and physics-based energy functions such as CHARMM36m (Huang *et al.*, 2016 ▸).

The next step is side-chain modelling, which involves the process of refining/adding side chains to the backbone built. Strategies such as dead-end elimination, Monte Carlo sampling and simulated annealing are usually used to sample the most probable rotamer (side-chain conformation), based on the local conformation of the backbone, from rotamer libraries such as that used by *SCWRL* (Krivov *et al.*, 2009 ▸). Once the model has been produced, it is usually refined to minimize un­favourable collisions between atoms. This is usually performed by performing energy minimizations following molecular-dynamics simulations using force fields. Excessive refinement may cause the model to deviate significantly from the original template (for some recent approaches, see Kim & Kihara, 2016 ▸; Park *et al.*, 2016 ▸; Lee *et al.*, 2016 ▸; Feig, 2016 ▸).

Following the introduction of *MODELLER*, many other approaches were developed for protein structure prediction. To assess their performance and to identify which features work best, an independent assessment initiative was established in 1994 (Moult *et al.*, 1995 ▸). The Critical Assessment of Protein Structure Prediction (CASP) is a community-wide experiment that is held biannually. Whilst CASP1 had only three categories (comparative modelling, fold recognition and *ab initio* modelling), many more categories have been introduced since then, such as accuracy of predictions for residue–residue contacts and disordered regions. Other categories include model-quality assessment, model refinement, data-assisted prediction, protein complex prediction and, recently, prediction of biological relevance. All of these categories are important in structural modelling (Moult *et al.*, 2016 ▸), and we highlight a few of them in this article, particularly those relating to recent developments in comparative modelling.

## 3. Recent developments in structural modelling   

Whilst it is outside the scope of this article to provide a historical review of developments in comparative modelling, we highlight some recent breakthroughs which have improved performance. An exciting recent development relates to more accurate predictions for residue–residue contacts. Residue-contact information has been used in the past, albeit not very successfully (*i.e.* with >80% of false positives; Monastyrskyy *et al.*, 2014 ▸), and whilst these approaches included co-evolution methods, performance was poor because it was difficult to separate indirect couplings from direct couplings. In addition, very sequence-diverse multiple sequence alignments were typically required. Recently, methods based on direct coupling analysis have been able to disentangle direct couplings from indirect couplings (Marks *et al.*, 2011 ▸; Jones *et al.*, 2012 ▸; Nugent & Jones, 2012 ▸; Kamisetty *et al.*, 2013 ▸). Furthermore, in some cases the problem of obtaining a sufficient number of diverse sequences can be solved by using metagenome data (Ovchinnikov *et al.*, 2017 ▸).

In addition, machine-learning approaches (recently deep learning) that utilize features related to the residue type (*i.e.* polarity *etc.*), structural characteristics (*i.e.* solvent exposure, secondary structure *etc.*), sequence separation length between the residues under consideration and pairwise information between all of the residues involved also show promise in contact prediction (Eickholt & Cheng, 2012 ▸; Feinauer *et al.*, 2014 ▸; Adhikari & Cheng, 2016 ▸).

The best residue-contact predictor in CASP11 (Monastyrskyy *et al.*, 2016 ▸) was *MetaPSICOV* (Jones *et al.*, 2015 ▸; Kosciolek & Jones, 2016 ▸), which integrates both co-evolution and machine-learning methods. Since then, many more structural groups have started to employ residue contacts using integrative methods (Skwark *et al.*, 2014 ▸; He *et al.*, 2017 ▸) or deep-learning methods (Wang *et al.*, 2017 ▸), ultimately using these data to guide three-dimensional structure modelling. In the template-free category of CASP11, an accurate structural model of a 256-residue protein was successfully generated by incorporating contact information (Monastyrskyy *et al.*, 2016 ▸). In addition, residue-contact data can be used for model ranking, selection, evaluation and refinement (Adhikari & Cheng, 2016 ▸; Park *et al.*, 2016 ▸).

Other recent developments are the application of different profile-based methods in template identification and sequence alignment [Markov random fields (Ma *et al.*, 2014 ▸) and conditional random forests (Joo *et al.*, 2016 ▸)], the use of integrated template-based and *ab initio* approaches (Yang *et al.*, 2016 ▸), and better methods for protein model refinement with improved energy functions and MD simulations (Kim & Kihara, 2016 ▸; Park *et al.*, 2016 ▸; Lee *et al.*, 2016 ▸; Della Corte *et al.*, 2016 ▸; Feig, 2016 ▸).

Below, we describe some recent developments in the methods from two structural modelling groups (the Lee group and the Zhang group) that performed consistently well in the template-based modelling category (based on the sum of *Z*-scores of different scoring measures) over the last few rounds of CASPs (CASP9, CASP10, CASP11 and CASP12; Mariani *et al.*, 2011 ▸; Huang *et al.*, 2014 ▸; Modi *et al.*, 2016 ▸).

The Lee group (Joo *et al.*, 2014 ▸, 2016 ▸; Joung *et al.*, 2016 ▸) follow the usual comparative modelling procedures. The modelling pipeline (*nns*) uses *FOLDFINDER*, an in-house method which utilizes profile–profile alignment and predicted secondary structures, *CRFpred*, another in-house conditional random-fields method, and *HHsearch* to search for structural templates. The sequence alignments are generated using *CRFalign* (Joo *et al.*, 2016 ▸), which is based on conditional random fields. *MODELLER* (main chain) is employed for the comparative modelling process. Side-chain modelling is performed by combining *SCWRL*4 (Krivov *et al.*, 2009 ▸) and an in-house residue-specific rotamer library. There is also a refinement step of the models using molecular-dynamics simulations.

In CASP12, the Lee group employed the new model-quality assessment method *SVMQA* to help with template selection and the model-quality assessment process (Manavalan & Lee, 2017 ▸). In addition, a new predicted residue–residue contact-based energy function (from *MetaPSICOV*) was added in the chain-modelling step. The success of the Lee group in CASP is largely owing to the use of an efficient global optimization method (finding the global minimum energy conformation for polypeptides) that is applied at different stages of modelling: sequence alignment, three-dimensional main-chain modelling and side-chain remodelling.

The Zhang group has also been a top contender in template-based modelling for the last few CASP rounds. The structural modelling of the Zhang group is based on *I-TASSER* (Yang *et al.*, 2015 ▸), an iterative fragment-based pipeline (threading). The *LOMETS* threading method is used to identify different structural fragments that are similar to the query structures (Wu & Zhang, 2007 ▸). The different fragments are then reassembled into full-length models using replica-exchange Monte Carlo simulations. Side-chain modelling is performed using *REMO* (Li & Zhang, 2009 ▸), which utilizes the *SCWRL* library (Krivov *et al.*, 2009 ▸). After this, the models are refined based on the free-energy states and at an atomic level using fragment-guided molecular-dynamics simulations (Zhang *et al.*, 2011 ▸). Finally, multiple model-quality assessment methods are used to select the best model.

A recent development is the implementation of *QUARK* (an in-house *ab initio*-based approach using small fragments of less than 20 residues; Xu *et al.*, 2012 ▸) into the *I-TASSER* pipeline. This new implementation was benchmarked in CASP11 (‘Zhang’ and ‘Zhang-Server’) and was shown to improve the overall quality of the models built compared with the *I-TASSER* pipeline without using *QUARK*. In CASP12, the Zhang group introduced *NN-BAYES*, a neural network and naïve Bayes classifier-based residue-contact predictor, into the *QUARK* protocol (He *et al.*, 2017 ▸). *NN-BAYES* collates the data from three machine-learning programs, three co-evolution programs and two metaservers: *MetaPSICOV* (Jones *et al.*, 2015 ▸) and *STRUCTH* (Sun *et al.*, 2015 ▸).

Although these two servers are among the top contenders in structural modelling, there are other highly ranked servers from CASP11 and CASP12 which the reader is advised to investigate (see CASP11 and CASP12 for access details; Modi *et al.*, 2016 ▸; http://predictioncenter.org/casp12/zscores_final.cgi). Reviewing all of these is outside the scope of this article. Most of the methods and servers assessed in CASP have been established to cope with individual queries or limited sets of sequences to be modelled, and none are dedicated to large-scale comparative modelling of genome sequences. In §[Sec sec4.1]4.1, we review some established resources and a more recent resource established to provide models for large numbers of genome sequences.

## 4. Model-quality assessment methods   

A good-quality protein model should resemble a native protein. Native proteins usually have compact, well packed three-dimensional structures. The spatial features of the residues should comply with empirically characterized constraints on torsional angles captured in Ramachandran plots (Ramachandran *et al.*, 1963 ▸). Hydrophobic side chains of the protein are buried to reduce unfavourable contacts with water molecules. Hydrogen bonds, disulfide bridges, salt bridges and covalent bonds should be present, as these facilitate the folding and packing of the polypeptide chain.

The methods typically used by structural biologists to check whether their crystal structures are well determined include *PROCHECK* (Laskowski *et al.*, 1993 ▸) and *MolProbity* (Chen *et al.*, 2010 ▸), which determine whether a protein structure has native-like features. These methods use various approaches to rule out unlikely protein structures with unfavourable stereochemical properties such as Ramachandran outliers, steric clashes, incorrect hydrogen bonds and distorted bond angles.

From a thermodynamic perspective, native proteins are always folded in the lowest energy state (Rangwala & Karypis, 2010 ▸). Many energy-based programs have been developed to select the most native-like model, with the lowest energy state, from decoy sets. Statistical potential energy-based functions are derived from statistical analysis of the growing numbers of experimental protein structures. In contrast, physics-based energy functions use molecular-mechanics force fields of molecules that take into account bond lengths, torsion angles, van der Waals forces and electrostatic interactions (Brooks *et al.*, 1983 ▸; Weiner *et al.*, 1984 ▸; Scott *et al.*, 1999 ▸).

In addition, the quality of protein models can also be assessed by checking the compatibility of the models produced with the conservation of the sequence pattern. The core of the proteins is usually composed of conserved residues. In contrast, protein surface residues tend to be less conserved, with more variability (Branden & Tooze, 1999 ▸).

The current state-of-the-art model-quality assessment methods can be divided into two main types: single-model methods and clustering methods.

### 4.1. Single-model methods   

Single-model methods use evolutionary information (Kalman & Ben-Tal, 2010 ▸), statistical potentials, physics-based potentials and combinations of different features (Benkert *et al.*, 2011 ▸; Cao & Cheng, 2016 ▸; Singh *et al.*, 2016 ▸; Liu *et al.*, 2016 ▸) obtained from only one model to evaluate the model quality (Wallner & Elofsson, 2003 ▸).

The most commonly used statistical potential-based model-quality assessment method is *MODELLER*’s *DOPE* score (Shen & Sali, 2006 ▸). *DOPE* is an atomic distance-dependent statistical potential based on a physical reference state that accounts for the finite size and spherical shape of proteins. Other statistical potential methods are also available. They differ in the sample set of known protein structures used, the protein representation (*e.g.* all atoms, C^α^ atoms), the spatial features (*e.g.* angles, distances, solvent accessibility, inter­atomic contact areas) and the definition of the reference state (Dong *et al.*, 2013 ▸). Recently, new methods such as *GOAP* (Zhou & Skolnick, 2011 ▸), *SOAP* (Dong *et al.*, 2013 ▸), *DOOP* (Chae *et al.*, 2015 ▸) and *VoroMQA* (Olechnovič & Venclovas, 2017 ▸) have been introduced and all have claimed to be more reliable than their counterparts.

Model-quality assessment methods exploiting machine-learning (ML) methods are also becoming popular. The major advantage of ML methods is their ability to take a large number of features into account simultaneously, often capturing the hidden relationships among them, which are hard to deduce using energy-term measures alone. *ProQ*2 combines evolutionary information, multiple sequence alignment data and structural features from the model using a support vector machine (SVM) to assess the quality (Ray *et al.*, 2012 ▸). The recent *ProQ*3 uses a deep-learning method to combine *ProQ*2 with *Rosetta* energy terms (Leaver-Fay *et al.*, 2011 ▸) and has been shown to be superior to *ProQ*2 (Uziela *et al.*, 2016 ▸, 2017 ▸). *DeepQA* is another deep-learning method that combines physiochemical properties (*i.e.* secondary-structure similarity and solvent accessibility) and statistical potential energy terms (Cao *et al.*, 2016 ▸). *MQAPRank* is a machine-learning-to-rank method that extracts features from statistical potentials and the scores obtained from a few model-quality assessment methods (Jing *et al.*, 2016 ▸). *SVMQA* is an SVM method that combines eight statistical potential energy terms and 11 consistency-based terms (between the predicted values from the sequence of the query protein and the calculated values from the model built; Manavalan & Lee, 2017 ▸).

Besides assessing the model from a global perspective, local quality assessments of protein models are also available. It is possible to discriminate between good/bad modelled regions of a whole protein chain using software such as *QMEAN* (Benkert *et al.*, 2008 ▸), *ProQ*2 (Ray *et al.*, 2012 ▸) and *ModFOLD* (Maghrabi & McGuffin, 2017 ▸).

### 4.2. Clustering methods   

In contrast to single-model methods, clustering methods are based on the structural comparison of multiple models generated for a single target. All-against-all structural comparisons are first carried out and the resulting scores are used to generate an *N*-dimensional distance matrix based on the structural distances between each model.

These approaches assume that the best model is the model structure with the lowest average distance to the rest of the data set (Konopka *et al.*, 2012 ▸). Therefore, after clustering the models these approaches select the centroid for each cluster. The best model of the whole decoy data set usually lies within the largest structurally conserved cluster. A model-quality score for the model is calculated by averaging the structural comparison scores obtained from all pairwise comparisons (model *versus* model) within the cluster and is usually followed by normalization of the score. Recent methods that use clustering approaches include *PconsD* (Skwark & Elofsson, 2013 ▸), *MULTICOM-CONSTRUCT* (Cao *et al.*, 2014 ▸) and *ModFOLD6_rank*/*ModFOLD6_cor* (Maghrabi & McGuffin, 2017 ▸).

### 4.3. Recent developments in model-quality assessment   

Model-quality assessment by clustering has typically been superior to other quality-assessment methods. However, these approaches fail to identify good-quality models if the majority of the models are of bad quality and are structurally similar to each other. The other problem with clustering methods is their high computational cost.

Furthermore, there have recently been many single-model methods that can achieve better performance than clustering methods, for example in the CASP category that selects good-quality models from decoys (http://predictioncenter.org/casp12/qa_diff2best.cgi). This is probably owing to the rise of machine-learning methods. *SVMQA* is an SVM method that is based on the combination of two independent predictors trained on the TM score or GDT_TS score (Manavalan & Lee, 2017 ▸). Other methods exploit deep learning and machine-learning-to-rank, which seem to be superior to SVMs (Uziela *et al.*, 2016 ▸, 2017 ▸; Cao *et al.*, 2016 ▸; Jing *et al.*, 2016 ▸).

## 5. Resources dedicated to large-scale comparative modelling of genome sequences   

As mentioned above, there have been several recent developments in comparative modelling, and many excellent servers are now available for biologists wishing to model the structure of a query protein [for more information on the servers that are currently highly ranked, see Modi *et al.* (2016 ▸) or http://predictioncenter.org/casp12/zscores_final.cgi]. Therefore, for the remainder of this article, since the focus in our group is more related to providing libraries of structural templates and a library of structural models, we consider resources providing large repositories of pre-calculated three-dimensional models. The methods used to generate these repositories have either not been regularly assessed by CASP or do not currently rank top in CASP [although some, for example *Phyre*2 (Kelley *et al.*, 2015 ▸) and *pGenThreader* (Lobley *et al.*, 2009 ▸) have had overall good rankings for over 20 years]. However, they have been applied to generate large or very large libraries of models and can therefore be useful for larger-scale requests from biologists.

In particular, we focus on four resources that provide pre-calculated three-dimensional structural models for over 100 000 UniProt sequences (for multiple model organisms) and for each we describe how the structural models are built. These resources provide easy access to three-dimensional structure data, visualize these structures using state-of-the-art visualization platforms and also provide functional annotations, where available, for example inherited binding-site information and other information valuable for life-science researchers.

### 5.1. ModBase   

ModBase (Pieper *et al.*, 2014 ▸) was developed by the Sali group in 1998 and currently contains more than 36 000 000 protein models (5 956 279 unique sequences) from at least 66 species (as of April 2017). ∼82% of the 170 418 human transcripts in the database are annotated with structural models. ModBase uses *ModPipe* (Eswar *et al.*, 2003 ▸), an automated pipeline, to produce the models. *ModPipe* utilizes a whole range of template-selection methods (sequence–sequence, sequence–profile, profile–profile), including *PSI-BLAST* and *HHsearch*. The alignment obtained from the template-selection method is then fed into *MODELLER* for the modelling process. *MODELLER* is based on the satisfaction of spatial restraints theory inspired by NMR spectroscopy. These restraints include homology-derived restraints obtained from the alignment of query sequences and template structures, stereochemical restraints extracted from the CHARMM22 molecular force field (Brooks *et al.*, 1983 ▸) and statistical restraints compiled from a list of known protein structures. Based on the alignment between the query and the model, a set of spatial restraints are derived, which include bond distances, bond angles, dihedral angles and van der Waals repulsions. These are expressed as probability density functions, which are combined into an objective function used to calculate the location of each atom in the protein (Sali & Blundell, 1993 ▸). For each model ModBase provides five different quality-assessment criteria [sequence identity, GA341 (Melo *et al.*, 2002 ▸), normalized *DOPE* score (Shen & Sali, 2006 ▸), *ModPipe* Quality Score and *TSVMod* score (Eramian *et al.*, 2008 ▸)].

In addition to the model quality, the target–template alignment and sequence identity are also provided. In addition, some of the entries contain information about putative ligand-binding sites, SNP annotation and protein–protein interactions.

### 5.2. The SWISS-MODEL repository   

SWISS-MODEL (Bienert *et al.*, 2017 ▸) is another comprehensive repository providing three-dimensional structural models for the 12 most accessed genomes in UniProtKB. It houses more than 900 000 models for UniProt sequences. Of the 21 042 human sequences, ∼75% are annotated with at least one structural model. The SWISS-MODEL repository also provides structural models for homo-oligomeric complexes. All of the homology models were created using the in-house modelling platform *PROMOD*3 (Bienert *et al.*, 2017 ▸), which uses *BLAST* and *HHsearch* for template searching. In order to facilitate oligomeric complex modelling, structural templates in the database are also organized as quaternary-structure assemblies. The database is updated weekly and contains more than ∼81 000 unique sequences in ∼180 000 assemblies. *QMEAN* (Benkert *et al.*, 2008 ▸) is used to assess the quality of the models. As well as model quality, all models are provided with the target–template alignment and sequence identity. Some of the entries contain InterPro functional annotations (Finn *et al.*, 2017 ▸). SWISS-MODEL plans to model more homo-oligomeric complexes, even for distant relatives, and to possibly include hetero-oligomeric complexes.

### 5.3. The Protein Model Portal   

The Protein Model Portal is a database which collects both experimental structures and structural models. As well as structural models found in the ModBase and SWISS-MODEL repositories, models generated by some of the NIH-funded Protein Structure Initiative (PSI) centres are also included. Based on UniProt release 2017_1, the portal comprises 5 388 221 unique sequences covered by at least one model. By combining models from different resources, the suppliers of the Protein Model Portal can apply the same model-quality assessment and validation criteria to them. Again, each model is provided with the sequence–template alignment and sequence identity. The user can also request further assessment of model quality, as the portal provides a submission interface to other quality-assessment servers such as *ModEval* (Eramian *et al.*, 2008 ▸), *QMEAN* (Benkert *et al.*, 2009 ▸) and *ModFOLD* (Maghrabi & McGuffin, 2017 ▸). Furthermore, the models provided by different resources can be structurally superposed to analyse the variability amongst them. For any queries with no currently available structural model, the portal provides a submission interface to modelling servers such as *I-TASSER* (Yang *et al.*, 2016 ▸) and *Phyre*2 (Kelley *et al.*, 2015 ▸).

### 5.4. The Genome3D initiative   

Genome3D (Lewis *et al.*, 2015 ▸) is a UK-based collaborative project to annotate genome sequences with structural information. The participating partners includes *Gene*3*D* (Lam *et al.*, 2016 ▸), *SUPERFAMILY* (Wilson *et al.*, 2009 ▸), *Phyre*2 (Kelley *et al.*, 2015 ▸), *VIVACE* (Ochoa-Montaño *et al.*, 2015 ▸), *pDomTHREADER* (Lobley *et al.*, 2009 ▸) and *BioSerf* (Buchan *et al.*, 2013 ▸). Each resource provides models based on either SCOP or CATH domain structures. Therefore, to facilitate the comparison of predicted models, Genome3D identifies matching CATH and SCOP superfamily pairs. Genome3D uses both homology-based approaches (*Gene*3*D*, *SUPERFAMILY* and *Phyre*2) and threading-based approaches (*FUGUE*, *pDomTHREADER* and *Phyre*2) to provide structural annotations for UniProt sequences. Genome3D annotates 94.6% of the 20 195 human sequences with at least one structural domain annotation. In addition to this, 88% of the 20 195 human sequences are annotated with three-dimensional structural models. Structural models in the resource were built by the following comparative modelling and threading methods.

*BioSerf* (Buchan *et al.*, 2013 ▸) is a fully automated pipeline that combines comparative modelling, protein threading and *ab initio* approaches. *BioSerf* searches for a suitable homologous template using *PSI-BLAST* and *HHsearch*. *MODELLER* is then used to build the model. Protein threading is performed using the in-house threading methods *pGenTHREADER* (Lobley *et al.*, 2009 ▸) and *pDomTHREADER* (Lobley *et al.*, 2009 ▸) guided by the protein secondary-structure prediction method *PSIPRED* (Jones, 1999 ▸). The *FRAGFOLD* algorithm is used, where appropriate, to create *ab initio* models. *FRAGFOLD* uses known protein super-secondary-structural fragments and uses a simulated-annealing algorithm to assemble the most probable three-dimensional protein structure (Kleywegt & Jones, 1997 ▸). Recently, the Jones group introduced *EigenTHREADER*, a novel fold-recognition method which combines standard threading methods with their in-house *MetaPSICOV* contact-prediction constraints method (Buchan & Jones, 2017 ▸).

*Phyre*2 (Kelley *et al.*, 2015 ▸) relies on *HHsearch* searches. Once templates have been identified, *MODELLER* is then used to predict the most probable model. Amino-acid side chains are added to the final model using *SCRWL* (Krivov *et al.*, 2009 ▸). In addition to the comparative modelling pipeline, *Phyre*2 also provides multiple-template and *ab initio* approaches to model the query. Recently, *Phyre*2 introduced *PhyrePower*, which models queries with distant homology using contact threading, *i.e.* pairwise alignment of eigen­decomposed contact maps (https://hub.docker.com/r/filippis/phyrepower-docker/). *VIVACE* (Ochoa-Montaño *et al.*, 2015 ▸) uses the *FUGUE* environment-specific substitution table and structure-dependent gap-penalty homology-detection method (Shi *et al.*, 2001 ▸) to search for structural templates from the TOCCATA library (B. Ochoa-Montaño, R. Bickerton & T. L. Blundell; http://structure.bioc.cam.ac.uk/toccata). If several structural templates are matched, they are aligned using *BATON* (a streamlined version of *COMPARER*; Sali & Blundell, 1990 ▸). *VIVACE* uses the sequence-alignment module (which uses information from multiple sequences and structures) implemented in *FUGUE* (for further details, see Shi *et al.*, 2001 ▸) to align the query with the template. Subsequently, the alignment is fed into *MODELLER* to generate a model. Both *SUPERFAMILY* and *Gene*3*D* use *HMMer*3 (Eddy, 2011 ▸) to search their template libraries (based on SCOP and CATH, respectively). Structural models are created by using the HMM alignment of the sequence to the best superfamily and are then resolved using *MODELLER*.

## 6. Improvements in template selection obtained by subclustering protein domain superfamilies   

As mentioned in §[Sec sec2.1]2.1, several approaches are used to identify a close relative with known structure for use as a template for comparative modelling. Where very close homologues are available (≥40% sequence identity), it is possible to detect the closest template using the results returned by *BLAST*. However, when only remote homologues are available it is best to scan against sequence profiles or HMMs constructed from closely related sets of homologues, for example within a SCOP or CATH superfamily. The Orengo group recently developed a subclassification of CATH protein domain superfamilies that clusters relatives that are likely to have very similar structures and functions.

Functional families (FunFams) were introduced as a subclassification of superfamilies inside CATH-Gene3D, a resource which provides evolutionary classification of structures and sequences for known protein domains (Lam *et al.*, 2016 ▸; Dawson *et al.*, 2017 ▸). When FunFams were used to select templates for building models of structurally uncharacterized relatives in 11 large, structurally and functionally diverse superfamilies in the Structure Function Linkage Database (SFLD; Akiva *et al.*, 2014 ▸), the structural coverage of models was up to five times greater, for some superfamilies, compared with selecting targets using a 30% sequence-identity cutoff. Furthermore, despite the fact that many remote homologues needed to be used as templates, these models were found to be of similar quality to those built using close sequence homologues (≥30% sequence identity) as parents (Lee *et al.*, 2010 ▸).

A recent, more accurate FunFam identification protocol (*FunFHMMer*; Das *et al.*, 2015 ▸) uses similarities in sequence patterns, reflecting highly conserved positions and specificity-determining positions, to guide subclustering and family detection. Highly conserved positions are generally important for the stability, folding or function of the protein domain. Specificity-determining positions are positions that are conserved within and unique to a particular cluster, sharing a specific function and usually involved in functional divergence from other clusters (Abhiman & Sonnhammer, 2005 ▸; Rausell *et al.*, 2010 ▸).

**Figure 1:**
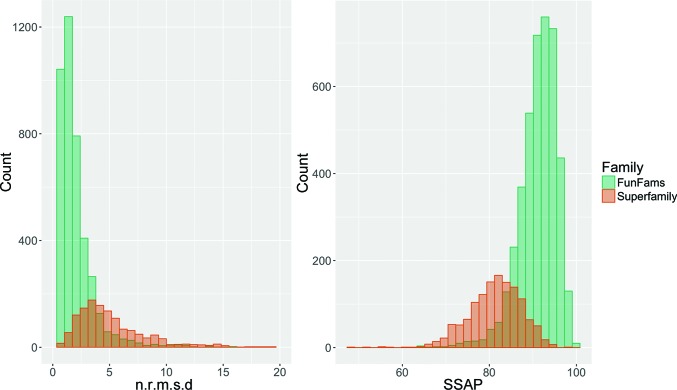
Structural conservation of structural domains classified in CATH FunFams and superfamilies.

Functional purity of the new FunFams was demonstrated in a number of ways: by validating against experimentally determined Enzyme Commission (Webb, 1992 ▸) and SFLD (Akiva *et al.*, 2014 ▸) annotations and also by checking whether known functional sites coincide with highly conserved residues in the multiple sequence alignments of FunFams (Das *et al.*, 2015 ▸). Functional predictions based on FunFams were ranked amongst the top five methods for the ‘Molecular Function’ category and the ‘Biological Process’ category in the Second CAFA International Function Prediction experiment (Jiang *et al.*, 2016 ▸). It can also be seen from Fig. 1 ▸ that relatives within FunFams tend to be much more structurally conserved than relatives across the whole superfamily. To generate this plot, we clustered all structural domains for each FunFam into sequence-identity 90% (S90) clusters. A representative was selected with a length that was closest to the average length of domains in the cluster and with the best X-ray resolution. Pairwise structural comparisons between representatives were performed using the *SSAP* structure-comparison algorithm (Taylor & Orengo, 1989 ▸). We also compared across superfamilies, comparing representatives from 35% sequence identity (S35) clusters, selecting representatives in the same way, again using *SSAP* to compare them. We took the mean of normalized r.m.s.d. (n.r.m.s.d.) and *SSAP* score for the comparisons. The r.m.s.d. values were normalized based on the larger of the two domains being compared.

Most pairs of FunFam domains have an n.r.m.s.d. difference between 0 and 5 Å and an *SSAP* score between 80 and 100 (the range is 0–100). By contrast, for pairs of superfamily domains the n.r.m.s.d. values have a wider spread from 0 to 10 Å and the *SSAP* score differences are between 70 and 90. The SSAP score and n.r.m.s.d. differences between the groups were statistically significant (*p*-value < 2.2 × 10^−16^; Mann–Whitney *U* test), demonstrating greater structural conservation within FunFams.

### 6.1. Assessment of CATH FunFams in template selection   

The significant structural coherence of the FunFams suggested that FunFams might be a reasonable classification level for selecting templates for comparative modelling. To test their value in template selection, we compared their performance against the well established template-selection method *HHsearch* employed by most of the successful structural modelling groups in recent CASPs, such as *Robetta* (Kim *et al.*, 2004 ▸; Ovchinnikov *et al.*, 2017 ▸), *MULTICOM* (Li *et al.*, 2015 ▸) and *nns* (Joo *et al.*, 2016 ▸).

*HHsearch* scans query sequences against a library of HMMs (built using *HHsuite*) and outputs a list of structural matches and corresponding query–template matches. Our FunFams pipeline first assigns a query sequence to a FunFam using *HMMer*3 (Eddy, 2011 ▸) and then selects the best template from the FunFam based on the sequence identity (the *E*-value should be <0.01) and X-ray resolution. For the *HHsearch* pipeline, we used *HHsearch* to scan for the best template, which was selected using the program’s built-in statistical measures (*E*-value and probability of being a true positive). After this, for both modelling strategies we employed *HHsearch* to generate the query–template alignments, and *MODELLER* v.9.15 was then used to predict ten models for each query target for each template-selection method. The best model was selected based on *MODELLER*’s built-in statistical potential: the *DOPE* score. The quality of the selected final three-dimensional models was assessed using the sequence-dependent structural superposition program *TMscore* (Zhang & Skolnick, 2004 ▸; Xu & Zhang, 2010 ▸), which superposed the three-dimensional model against the native protein structure. A benchmark data set of 8633 non­redundant CATH close-homologue targets (query targets that have sequence relatives with ≥30% global sequence identity) and 602 remote-homologue targets (query targets that have sequence relatives with <30% global sequence identity) were used.

Overall, FunFams gave higher percentages of good models compared with *HHsearch* for both close homologues [96.4% (*HHsearch*) *versus* 98.2% (FunFams)] and remote homologues [76.6% (*HHsearch*) *versus* 93.8% (FunFams), *p*-value < 1 × 10^−19^; Mann–Whitney *U* test]. The results of our assessment suggest that it is helpful to subclassify homologues according to likely structural and functional similarity prior to performing template selection. A comparative modelling platform that uses both the FunFam and *HHsearch* template-searching algorithms has been developed to provide three-dimensional models for *Gene*3*D* and Genome3D. Structural models have been built for the human (at least one domain for 72% of the sequences) and fly (at least one domain for 70% of the sequences) genomes. These are currently available from the *Gene*3*D* resource (Lam *et al.*, 2016 ▸).

### 6.2. Assessment of CATH FunFams in template selection (modelling binary protein–protein interactions)   

Since large-scale functional genomics data are accumulating and suggest the value of systems-based approaches for understanding the biological role of a protein, we also explored the performance of FunFams in modelling binary protein–protein interactions (*i.e.* complexes) using *MODELLER*. To perform this, we used query sequences from a publicly available benchmark data set of structures used by the Interactome3D resource to provide complexes for their November 2011 release (Mosca *et al.*, 2013 ▸), which could be mapped to CATH. This allowed us to compare our results with those reported in Mosca *et al.* (2013 ▸), who used *BLAST* to select templates, followed by *MODELLER* to model complexes, for the same data set. We also built models for a publicly available benchmark sequence set in the May 2015 release of Interactome3D, which could be mapped to CATH domains. The *BLAST*-based protocol reported in Mosca *et al.* (2013 ▸) only builds models if there is a structural template from a close homologue with a minimum global sequence identity of 40%. We selected protein–protein interactions (PPIs) for which the query PPIs had been classified in CATH and a structural template could be found for both chains. The PPI sequence subset modelled by FunFams was slightly more difficult overall than the set modelled by the *BLAST* protocol, as a quarter of the query targets share a sequence identity of <40% with the closest template.

**Figure 2:**
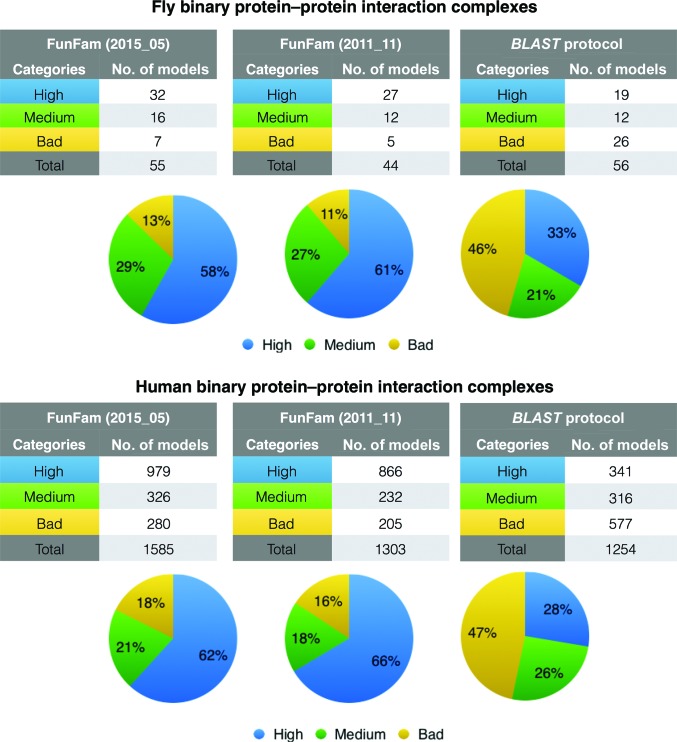
Comparison of the quality of the top-ranked models produced by modelling protocols using functional families (FunFams) and a *BLAST*-based strategy. The models were assessed by perfoming a structural comparison with the known protein complexes. We used the assessment criteria adopted by the Critical Assessment of Prediction of Interactions (CAPRI) to classify the models into different categories based on the interface r.m.s.d. (i.r.m.s.d.) and fraction of native residue–residue contacts (Fnat) (Méndez *et al.*, 2003 ▸).

We found a significant improvement in model quality using templates selected by the FunFam protocol compared with a *BLAST* strategy (see Fig. 2 ▸). For the FunFam protocol, 89% and 84% of the fly and human binary PPIs are associated with medium- or high-quality models. In contrast, the top-ranked models produced by the *BLAST* strategy were medium to high quality for only 55% and 52% of the fly and human interactions, respectively. The FunFam protocol managed to produce 30% more medium/high-quality models than a protocol based on *BLAST*. Furthermore, a higher proportion of the models produced by the FunFam protocol (66% compared with 28%) are of high quality, again suggesting that it may also be valuable to use a functional family-based protocol to guide template selection in binary protein–protein interaction modelling.

## 7. Uses of structural modelling in experimental studies   

Below, we highlight a few selected examples of recent developments in techniques that exploit comparative models to improve the structural determination or structural coverage of large-scale macromolecular assemblies.

### 7.1. Facilitation of cryo-EM density map fitting with homology models   

New developments in cryo-electron microscopy (cryo-EM) have meant that this approach is increasingly used for the protein structure determination of large macromolecular complexes and assemblies. One major problem with cryo-EM is the low resolution of the density maps that are produced. To help with the interpretation of these density maps, they are usually fitted onto experimentally solved structures. However, owing to the low number of solved structures, it can sometimes be hard to find a suitable template. In 2005, the Topf group demonstrated that it is feasible to use comparative models for the fitting process. They subsequently developed a web server named *CHOYCE* (Rawi *et al.*, 2010 ▸) which performs homology modelling (*MODELLER*) and fitting into cryo-EM maps. The server allows the user to select the most accurate models (based on the *DOPE* score).

For those adventurous users who prefer to perform the modelling manually, Allen and Stokes exemplified the steps involved from building the structural models to the fitting of models to the density map using an integral membrane protein, CopA. In addition to this, they also illustrated how to dock additional components into the models using a computational approach (Allen & Stokes, 2013 ▸).

*Gorgon* (Baker *et al.*, 2016 ▸) can model not only a protein structure but entire macromolecular assemblies. For example, the C^α^ backbone model for every protein component in the ribosome (from an ∼4.5 Å resolution cryo-EM map) was automatically built in less than a day. *Gorgon* uses *ab initio* modelling, feature extraction and rigid-body and flexible fitting for model building. It also includes the use of statistical measures to evaluate the fit of an atomic model to the cryo-EM density map.

### 7.2. Integrative structural biology   

Integrative structural biology is a new field which tries to determine the three-dimensional structures of proteins by using the ensembles produced by experimental methods and computational approaches (Ward *et al.*, 2013 ▸). This is especially useful for proteins that are not crystallizable, are in­soluble, are too large or too small or are conformationally heterogeneous (Sali *et al.*, 2015 ▸).

Shi and coworkers used a refined integrative method that combines information generated from electron microscopy, X-ray crystallography and comparative structure modelling to provide a clear structural view of the Nup84 nucleoporin complex. This complex is a stable heteroheptameric (seven nucleoporins) protein complex of ∼600 kDa from budding yeast (Shi *et al.*, 2014 ▸).

Another interesting example is the structure of human prolactin receptor solved by Bugge and coworkers in 2016. This was the first ever full view of a class I cytokine receptor. Class I cytokine receptors are generally considered to be key drug targets. The comparative modelling tool *MODELLER* was employed to integrate structural data from NMR spectroscopy, small-angle X-ray scattering and native mass spectrometry to generate a structural model of the receptor. The structural model was generated by assembling all of the individual domains of the structure as overlapping segments (Bugge *et al.*, 2016 ▸).

## 8. Concluding remarks   

The last few years have been an exciting era for the protein structural modelling community. There have been substantial improvements in residue-contact prediction thanks to the use of direct coupling analysis, better statistical machine learning and the huge amount of new sequence data that is being provided by metagenome analyses. Many groups are now employing residue-contact prediction to enhance the performance of their methods. Better profile methods such as conditional random forest and Markov random fields have improved the accuracy of the template-selection process. In addition, we have demonstrated the value of organizing domain superfamilies into functional families (CATH FunFams) for template selection. CATH FunFams group relatives that are highly likely to be of similar structure and function. They are generated using a new functional sub­classification in CATH-Gene3D, which constrains clustering of relatives by ensuring that any new relatives joining a particular cluster match the highly conserved functional determinants for that cluster (for example likely specificity-determining residues that influence the type of compounds bound or protein inter­actions). The improvement in accuracy for template selection relative to the HMM-based strategy used by *HHsearch* is therefore likely to be owing to the fact that the FunFam template-selection process only allows very remote relatives to be selected if they share the same or highly similar residues at key functional sites. Although *HHsearch* uses a powerful search strategy for remote homologues, there is no explicit constraint to ensure that equivalent functional residues are matched.

As well as improvements in residue-contact prediction, there have also been improvements in the structural refinement category, with improved energy functions and MD simulations (for a recent review on structural refinement, see Feig, 2017 ▸). There are also promising recent developments in template-free modelling (for a review, see Kc, 2016 ▸). Finally, there has been an increase in the performance of single model-based model-quality assessment methods, thanks to the use of integrated approaches and promising new approaches using deep learning.
